# Expression profiling and Ingenuity biological function analyses of interleukin-6- versus nerve growth factor-stimulated PC12 cells

**DOI:** 10.1186/1471-2164-10-90

**Published:** 2009-02-24

**Authors:** Dieter Kunz, Gaby Walker, Marc Bedoucha, Ulrich Certa, Pia März-Weiss, Beatrice Dimitriades-Schmutz, Uwe Otten

**Affiliations:** 1 Department of Biomedicine, Institute of Physiology, University of Basel, Pestalozzistrasse 25, CH-4056 Basel, Switzerland; 2 Molecular Medicine Laboratories (MML), Hoffmann-La Roche Ltd., Grenzacherstrasse 2, CH-4002 Basel, Switzerland; 3 Discovery Research (PRBD), Hoffmann-La Roche Ltd., Grenzacherstrasse 2, CH-4002 Basel, Switzerland; 4 Non-Clinical Drug Safety (NCS), Hoffmann-La Roche Ltd., Grenzacherstrasse 2, CH-4002 Basel, Switzerland

## Abstract

**Background:**

The major goal of the study was to compare the genetic programs utilized by the neuropoietic cytokine Interleukin-6 (IL-6) and the neurotrophin (NT) Nerve Growth Factor (NGF) for neuronal differentiation.

**Results:**

The designer cytokine Hyper-IL-6 in which IL-6 is covalently linked to its soluble receptor s-IL-6R as well as NGF were used to stimulate PC12 cells for 24 hours. Changes in gene expression levels were monitored using Affymetrix GeneChip technology. We found different expression for 130 genes in IL-6- and 102 genes in NGF-treated PC12 cells as compared to unstimulated controls. The gene set shared by both stimuli comprises only 16 genes.

A key step is upregulation of growth factors and functionally related external molecules known to play important roles in neuronal differentiation. In particular, IL-6 enhances gene expression of regenerating islet-derived 3 alpha (REG3A; 1084-fold), regenerating islet-derived 3 beta (REG3B/PAPI; 672-fold), growth differentiation factor 15 (GDF15; 80-fold), platelet-derived growth factor alpha (PDGFA; 69-fold), growth hormone releasing hormone (GHRH; 30-fold), adenylate cyclase activating polypeptide (PACAP; 20-fold) and hepatocyte growth factor (HGF; 5-fold). NGF recruits GDF15 (131-fold), transforming growth factor beta 1 (TGFB1; 101-fold) and brain-derived neurotrophic factor (BDNF; 89-fold). Both stimuli activate growth-associated protein 43 (GAP-43) indicating that PC12 cells undergo substantial neuronal differentiation.

Moreover, IL-6 activates the transcription factors retinoic acid receptor alpha (RARA; 20-fold) and early growth response 1 (Egr1/Zif268; 3-fold) known to play key roles in neuronal differentiation.

Ingenuity biological function analysis revealed that completely different repertoires of molecules are recruited to exert the same biological functions in neuronal differentiation. Major sub-categories include cellular growth and differentiation, cell migration, chemotaxis, cell adhesion, small molecule biochemistry aiming at changing intracellular concentrations of second messengers such as Ca2+ and cAMP as well as expression of enzymes involved in posttranslational modification of proteins.

**Conclusion:**

The current data provide novel candidate genes involved in neuronal differentiation, notably for the neuropoietic cytokine IL-6. Our findings may also have impact on the clinical treatment of peripheral nerve injury. Local application of a designer cytokine such as H-IL-6 with drastically enhanced bioactivity in combination with NTs may generate a potent reparative microenvironment.

## Background

Interleukin-6 (IL-6) is the prototype member of the IL-6 cytokine family, also termed neuropoietic cytokines, including IL-6, IL-11, IL-27, ciliary neurotrophic factor (CNTF), leukemia inhibitory factor (LIF), oncostatin M, cardiotrophin-1 (CT-1), cardiotrophin-like cytokine (CLC; also known as novel neurotrophin 1, NNT1), neuropoietin and B cell stimulatory factor 3 (BSF3) [1,2]. A common feature of all family members is the signaling through a specific receptor that is associated to the intracellularly located transduction component gp130. Subsequently, the Janus-activated kinase-signal transducer, activator of transcription (JAK-STAT) and mitogen-activated protein kinase (MAPK) signal transduction pathways are activated. Neuropoietic cytokines display multiple functions in the peripheral (PNS) and central nervous systems (CNS), including the developing and adult brain, synaptic plasticity as well as the brain's response to injury and disease. In particular these molecules control cell fate and differentiation of neural stem and progenitor cells during development; due to their neurotrophic and regenerative actions they crucially affect injury-induced neurogenesis, neuronal survival and regeneration; moreover, these molecules can also influence neuronal activity and are implicated in long-term potentiation (LTP; reviewed in [2]).

Cellular functions of IL-6 are mediated by two specific receptors, the membrane-bound 80 KDa IL-6 receptor (IL-6R) or the soluble form of IL-6R (s-IL-6R) which can be generated either by shedding of IL-6R or by alternative splicing of the IL-6R mRNA [3,4]. Using s-IL-6R, IL-6 responsiveness may be conferred to cells expressing the transduction component gp130, but are devoid of membrane-bound IL-6R in the process of transsignaling [5-7]. The transsignaling mechanism led to the development of a fusion protein in which IL-6 is covalently linked to s-IL-6R thereby creating a unimolecular protein with enhanced biological activities. The fusion protein, termed Hyper-IL-6 (H-IL-6), turned out to be fully active at 100–1000-fold lower concentrations as compared to the combination of the two separate molecules [8,9].

The neurotrophin (NT) family of growth factors including nerve growth factor (NGF), brain-derived neurotrophic factor (BDNF), neurotrophin-3 (NT-3) and NT-4/5 is important for development, maintenance and survival of many different cell types in the PNS and the CNS [10]. NTs are also involved in regulating adult neurogenesis [11,12], learning and memory [13,14]. NTs are synthesized as proNT precursors that may be processed to mature NTs intra- and extracellulary by specific proteases [15]. NTs exert their effects via two different types of cellular receptors: pan-neurotrophin receptor p75 (p75NTR) which binds all NTs with a similar affinity, and the family of high affinity tyrosine kinase receptors (Trk). The interactions of proNTs and NTs with the NT-receptors comprise a complex signaling system thus generating a broad variety of biological effects [16,17].

In the first report of IL-6 actions on neural cells rat pheochromocytoma cells (PC12), a well characterised cellular model for neuronal differentiation, were incubated for up to 6 days with B-cell stimulatory factor BSF-2/IL-6 thereby inducing significant neurite outgrowth [18]. PC12 cells that were differentiated either using irradiation [19] or the well-known hypoxia mimetic agent CoCl2 [20] require IL-6 expression. We have demonstrated that primary sympathetic neurons [21] and PC12 cells [22] can strongly respond to IL-6 by transsignaling, and that the potential of IL-6 to induce neuronal differentiation in PC12 cells is in close correlation to the availability of s-IL-6R [22,23]. PC12 cell differentiation is accompanied by enhanced expression of GAP-43 mRNA at 24 hours after stimulation with IL-6/s-IL-6R [22]. Moreover, we found that the fusion protein H-IL-6 is a highly active molecule in inducing survival of cultured sympathetic neurons, comparable to the effects of NGF [21,22]. Recently, IL6RIL6, a fusion protein in which IL-6 is directly linked to the extracellular domain of the IL-6 specific receptor, has been used for expression profiling studies in primary cultures of dorsal root ganglia. In these cells, IL6RIL6 strongly increases axonal network and expression of neural genes [24].

A significant problem in the clinical treatment of peripheral nerve injury is that the currently used therapeutic approaches do not allow complete neuronal recovery [25]. Mixtures comprising neuropoietic cytokines, glial cell-line derived neurotrophic factor ligands (GFLs) and NTs are being tested for the suitability to generate a microenvironment with a high reparative potential upon local administration at the site of the lesion [26].

In the present study we monitored changes in neuronal gene expression induced by incubation of PC12 cells for 24 hours with H-IL-6 as well as NGF, and compared the genetic programs utilized by these stimuli for neuronal differentiation.

## Results

### Overall changes in gene expression patterns in IL-6- and NGF-stimulated PC12 cells

Affymetrix Gene Chip U34A arrays were used to analyse global changes in gene transcripts using a cutoff in the change of gene expression of > 2-fold. In PC12 cells stimulated for 24 h with 10 ng/ml H-IL-6, we found 130 differently expressed genes as compared to unstimulated controls. Of them, 94 genes were upregulated with gene expression values from 2-fold to 1085-fold, whereas 36 genes were found to be downregulated in the range from -2-fold to -61-fold. The genes are further classified into major functional categories including cytokines (2 up-regulated/0 down-regulated), enzymes (20/8), G-protein coupled receptors (2/3), growth factors (7/1), ion channels (2/0), kinases (4/4), nuclear receptors (2/1), peptidases (3/1), phosphatases (0/2), transcription regulators (8/4), transmembrane receptors (5/0), transporters (8/3) and molecules with other functions (31/9; Table 1).

**Table 1:** List of gene set regulated by IL-6 in PC12 cells

**Gene**		**Accession no.**	**Fold change**	**Subcellular location**
**Cytokines**				
chemokine ligand 13	CXCL13	AF044196	43	Extracellular Space
chemokine ligand 10	CXCL10	U17035	7	Extracellular Space
				
**Enzymes**				
cytochrome P450, 4f16	CYP4F16	U39207	424	Cytoplasm
ceruloplasmin	CP	AF202115	191	Extracellular Space
peptidyl arginine deiminase, type III	PADI3	D88034	142	Cytoplasm
acyl-CoA synthetase, member 1	ACSL1	D90109	102	Cytoplasm
transglutaminase 1	TGM1	M57263	93	Plasma Membrane
nitric oxide synthase 2A	NOS2A	U03699	58	Cytoplasm
ornithine carbamoyltransferase	OTC	M11266	43	Cytoplasm
Similar to Lysophospholipase	LOC374569	AB009372	37	Unknown
trehalase	TREH	AF038043	35	Plasma Membrane
kynureninase	KYNU	U68168	25	Cytoplasm
nitric oxide synthase 3	NOS3	AJ011115	21	Cytoplasm
glycine amidinotransferase	GATM	U07971	14	Cytoplasm
guanine nucleotide binding protein, alpha z	GNAZ	U77485	14	Plasma Membrane
ST6 galactosamide alpha-2,6-sialyltranferase 1	ST6GAL1	M83143	14	Cytoplasm
aldo-keto reductase, 1C1	AKR1C1	BAA92883	12	Cytoplasm
myxovirus resistance 1	MX1	P20591	9	Nucleus
aldolase C	ALDOC	X06984	3	Cytoplasm
2',5'-oligoadenylate synthetase 1	OAS1	Z18877	3	Cytoplasm
protein disulfide isomerise, A2	PDIA2	AAC50401	3	Cytoplasm
RNA (guanine-7-) methyltransferase	RNMT	BAA82447	3	Nucleus
polymerase, alpha 2	POLA2	AJ245648	-2	Nucleus
steroid-5-alpha-reductase, alpha 1	SRD5A1	J05035	-2	Cytoplasm
aminolevulinate, delta-, synthase 2	ALAS2	D86297	-3	Cytoplasm
glutathione S-transferase A3	GSTA3	X78847	-3	Cytoplasm
UDP glycosyltransferase 8	UGT8	BC075069	-3	Cytoplasm
cell division cycle 42	CDC42	U37720	-4	Cytoplasm
cysteine dioxygenase, type I	CDO1	M35266	-4	Cytoplasm
ST8 alpha-2,8-sialyltransferase 3	ST8SIA3	X80502	-5	Cytoplasm
				
**G-protein coupled receptors**				
adrenergic receptor, alpha-2B	ADRA2B	M32061	26	Plasma Membrane
arginine vasopressin receptor 2	AVPR2	AAB87678	5	Plasma Membrane
vasoactive intestinal peptide receptor 1	VIPR1	M86835	-2	Plasma Membrane
cholinergic receptor, muscarinic 3	CHRM3	AB017656	-3	Plasma Membrane
cholinergic receptor, muscarinic 4	CHRM4	M16409	-10	Plasma Membrane
				
**Growth factors**				
regenerating islet-derived 3 alpha	REG3A	L10229	1084	Extracellular Space
regenerating islet-derived 3 beta	REG3B	S43715	672	Extracellular Space
growth differentiation factor 15	GDF15	AJ011970	80	Extracellular Space
platelet-derived growth factor alpha	PDGFA	M29464	69	Extracellular Space
nudix-type motif 6	NUDT6	AF188995	22	Extracellular Space
jagged 2	JAG2	U70050	5	Extracellular Space
hepatocyte growth factor	HGF	X84046	4	Extracellular Space
macrophage stimulating 1	MST1	X95096	-4	Extracellular Space
				
**Ion channels**				
glutamate receptor, ionotropic, delta 2	GRID2	U08256	91	Plasma Membrane
purinergic receptor P2X	P2RX2	Y10475	11	Plasma Membrane
				
**Kinases**				
fyn-related kinase	FRK	U02888	122	Nucleus
Janus kinase 2	JAK2	U13396	120	Cytoplasm
phosphatidylinositol 4-kinase beta	PI4KB	D84667	2	Cytoplasm
pim-3 oncogene	PIM3	AF086624	2	Unknown
fer tyrosine kinase	FER	X13412	-2	Cytoplasm
mitogen-activated protein kinase kinase 5	MAP2K5	U37462	-2	Cytoplasm
fibroblast growth factor receptor 1	FGFR1	S54008	-3	Plasma Membrane
activin receptor, type IIA	ACVR2A	S48190	-4	Plasma Membrane
				
**Nuclear receptors**				
retinoic acid receptor alpha	RARA	U15211	20	Nucleus
nuclear receptor, *C2	NR3C2	M36074	8	Nucleus
vitamin D receptor	VDR	J03630	-4	Nucleus
				
**Peptidases**				
complement component 1s	C1S	D88250	230	Extracellular Space
caspase 1	CASP1	U14647	40	Cytoplasm
proteasome subunit, alpha 1	PSMA1	M29859	5	Cytoplasm
kallikrein-related peptidase 8	KLK8	AJ005641	-5	Extracellular Space
				
**Phosphatases**				
pyruvate dehydrogenase phosphatase 2	PDP2	AF062741	-4	Cytoplasm
protein tyrosine phosphatase receptor D	PTPRD	U57502	-9	Plasma Membrane
				
**Transcription regulators**				
signal transducer and activator of transcription 1	STAT1	AF205604	579	Nucleus
Kruppel-like factor 6	KLF6	AF072403	249	Nucleus
HIV-1 Tat interacting protein	HTATIP	AAB18236	159	Nucleus
HIV enhancer binding protein 2	HIVEP2	D37951	65	Nucleus
upstream transcription factor 1	USF1	U41741	22	Nucleus
early growth response 1	EGR1	M18416	3	Nucleus
interferon regulatory factor 1	IRF1	M34253	3	Nucleus
signal transducer and activator of transcription 2	STAT2	AF206162	3	Nucleus
breast cancer 1	BRCA1	U36475	-2	Nucleus
D site of albumin promoter binding protein	DBP	J03179	-2	Nucleus
nuclear factor I/B	NFIB	Y07685	-2	Nucleus
transcription elongation factor A 2	TCEA2	D12927	-5	Nucleus
				
**Transmembrane receptors**				
oxidized low density lipoprotein receptor 1	OLR1	AB018097	587	Plasma Membrane
histocompatibility 2, Q region locus 10	H2-Q10	M31018	160	Plasma Membrane
insulin-like growth factor 2 receptor	IGF2R	NM_000876	39	Plasma Membrane
Fc fragment of IgG receptor IIa (CD32)	FCGR2A	M64368	16	Plasma Membrane
growth hormone receptor	GHR	Z83757	12	Plasma Membrane
				
**Transporters**				
cadherin 17	CDH17	X78997	273	Plasma Membrane
solute carrier family 6, member 3	SLC6A3	M80570	90	Plasma Membrane
nucleoporin 153kDa	NUP153	L06821	83	Nucleus
solute carrier family 9, member 2	SLC9A2	L11004	32	Plasma Membrane
cadherin 17	CDH17	L46874	13	Plasma Membrane
lipocalin 2	LCN2	X13295	9	Extracellular Space
syntaxin 4	STX4	L20821	3	Plasma Membrane
secretory carrier membrane protein 2	SCAMP2	AF295405	2	Cytoplasm
solute carrier family 12, member 5	SLC12A5	U55816	-3	Plasma Membrane
solute carrier family 30, member 2	SLC30A2	U50927	-5	Plasma Membrane
syntaxin 5	STX5	U87971	-8	Cytoplasm
				
**Others**				
regenerating islet-derived 1 alpha	REG1A	J05722	796	Extracellular Space
TIMP metallopeptidase inhibitor 1	TIMP1	L31883	210	Extracellular Space
calcitonin-related polypeptide beta	CALCB	M11596	195	Extracellular Space
fibrinogen gamma chain	FGG	J00734	164	Extracellular Space
trans-golgi network protein 2	TGOLN2	X53565	113	Cytoplasm
LIM and senescent cell antigen-like domains 1	LIMS1	AAA20086	94	Plasma Membrane
alpha-2-HS-glycoprotein	AHSG	M29758	80	Extracellular Space
ribosomal protein L3-like	RPL3L	AAC50777	60	Unknown
collagen, type IV, alpha 5	COL4A5	AB041350	59	Extracellular Space
parvalbumin	LOC4951	J02705	58	Unknown
YTH domain containing 1	YTHDC1	AF144731	39	Cytoplasm
growth hormone releasing hormone	GHRH	Z34092	31	Extracellular Space
annexin A1	ANXA1	M19967	29	Plasma Membrane
collagen, type XII, alpha 1	COL12A1	U57362	26	Extracellular Space
regenerating islet-derived 3 gamma	REG3G	L20869	24	Extracellular Space
adenylate cyclase activating polypeptide 1	ADCYAP1	S83513	20	Extracellular Space
heat shock protein 90 kDa, alpha B 1	HSP90AB1	S45392	20	Cytoplasm
luteinizing hormone beta	LHB	U25653	17	Extracellular Space
galectin 5	LGALS5	L36862	8	Extracellular Space
myocilin	MYOC	AF093567	8	Cytoplasm
prolactin family 8a81	PRL8A8	AB000107	8	Extracellular Space
troponin C type 2	TNNC2	J05598	8	Unknown
ribosomal protein L18a	RPL18A	X14181	7	Cytoplasm
fibrinogen beta chain	FGB	U05675	6	Extracellular Space
tropomyosin 3	TPM3	X72859	4	Cytoplasm
tubulin, beta	TUBB	AB011679	4	Cytoplasm
extracellular proteinase inhibitor	EXPI	X13309	3	Extracellular Space
growth associated protein 43	GAP43	M16736	3	Plasma Membrane
galectin 9	LGALS9	U72741	3	Extracellular Space
tubulin, alpha 4a	TUBA4A	M13444	3	Cytoplasm
BCL2-like 11	BCL2L11	AF136927	2	Cytoplasm
integrin alpha 7	ITGA7	X65036	-2	Plasma Membrane
syndecan 2	SDC2	M81687	-2	Plasma Membrane
zinc finger protein 260	ZNF260	U56862	-2	Nucleus
filamin C	FLNC	AF119148	-3	Cytoplasm
metallothionein 3	MT3	S65838	-3	Cytoplasm
arginine vasopressin	AVP	M25646	-4	Extracellular Space
fasciculation and elongation protein zeta 1	FEZ1	U63740	-4	Cytoplasm
crystallin, alpha B	CRYAB	U04320	-6	Nucleus
neurofascin	NFASC	U81036	-7	Plasma Membrane

Gene description names, gene symbols are from IPA Tool; accession numbers are from GenBank

In PC12 cells stimulated for 24 hours with 50 ng/ml NGF, we identified 102 differently expressed genes as compared to unstimulated controls. Of them, 71 genes were upregulated with gene expression values from 2-fold to 303-fold, whereas 31 genes were found to be downregulated by -2-fold to -20-fold. Major functional categories include enzymes (18 up-regulated/9 down-regulated), G-Protein coupled receptors (2/2), growth factors (3/1), ion channels (7/2), kinases (6/2), peptidases (4/1), phosphatases (2/1), transcription regulators (0/2), transmembrane receptors (1/0), transporters (9/2) and molecules with other functions (21/9; Table 2).

**Table 2:** List of gene set regulated by NGF in PC12 cells

**Gene**		**Accession no.**	**Fold change**	**Subcellular location**
**Enzymes**				
rat senescence marker protein 2A gene	SMP2A	X63410	303	Cytoplasm
myosin, heavy chain 3	MYH3	K03468	133	Cytoplasm
lecithin-cholesterol acyltransferase	LCAT	X54096	101	Extracellular Space
UDP glucuronosyltransferase 2, polypeptide A1	UGT2A1	X57565	63	Cytoplasm
contactin 4	CNTN4	U35371	44	Plasma Membrane
phosphodiesterase 4B,	PDE4B	J04563	37	Cytoplasm
gulonolactone (L-) oxidase	GULO	J03536	34	Cytoplasm
superoxide dismutase 3	SOD3	Z24721	28	Extracellular Space
fibronectin 1	FN1	X15906	28	Plasma Membrane
acetylcholinesterase	ACHE	S50879	28	Plasma Membrane
tryptophan hydroxylase 1	TPH1	X53501	24	Cytoplasm
aldo-keto reductase family 1, member C1	AKR1C1	BAA92883	10	Cytoplasm
guanine nucleotide binding protein, alpha z	GNAZ	U77485	9	Plasma Membrane
aminoadipate aminotransferase	AADAT	Z50144	5	Cytoplasm
phospholipase D2	PLD2	D88672	4	Cytoplasm
N-deacetylase/N-sulfotransferase 1	NDST1	M92042	3	Cytoplasm
phosphate cytidylyltransferase 2	PCYT2	AF080568	2	Cytoplasm
peptidylprolyl isomerase A	PPIA	M19533	-2	Cytoplasm
Rab geranylgeranyltransferase alpha	RABGGTA	L10415	-2	Unknown
glutathione S-transferase A3	GSTA3	X78847	-3	Cytoplasm
cytochrome P450, 4F4	CYP4F4	U39206	-3	Cytoplasm
3-hydroxyanthranilate 3,4-dioxygenase	HAAO	D28339	-3	Cytoplasm
stearoyl-Coenzyme A desaturase 2	SCD2	AB032243	-4	Cytoplasm
aldo-keto reductase family 1, member C3	AKR1C3	L32601	-6	Cytoplasm
myxovirus resistance 2	MX2	X52711	-10	Nucleus
serine dehydratase	SDS	M38617	-11	Cytoplasm
				
**G-protein coupled receptors**				
calcitonin/calcitonin-related polypeptide alpha	CALCA	V01228	136	Plasma Membrane
angiotensin II receptor 1	AGTR1	NM_009585	50	Plasma Membrane
cholinergic receptor, muscarinic 3	CHRM3	AB017656	-2	Plasma Membrane
parathyroid hormone receptor 1	PTHR1	M77184	-3	Plasma Membrane
				
**Growth factors**				
growth differentiation factor 15	GDF15	AJ011970	131	Extracellular Space
transforming growth factor beta 1	TGFB1	X52498	101	Extracellular Space
brain-derived neurotrophic factor	BDNF	X67108	89	Extracellular Space
neuregulin 1	NRG1	U02324	-3	Extracellular Space
				
**Ion channels**				
calcium channel, voltage-dependent, beta 2	CACNB2	M80545	90	Plasma Membrane
glutamate receptor, ionotropic, delta 2	GRID2	U08256	78	Plasma Membrane
sodium channel, voltage-gated, type II, beta	SCN2B	U37147	73	Plasma Membrane
potassium inwardly-rectifying channel J4	KCNJ4	X87635	51	Plasma Membrane
solute carrier family 9 member 3	SLC9A3	M85300	40	Plasma Membrane
purinergic receptor P2X, ligand-gated ion channel 2	P2RX2	Y10475	13	Plasma Membrane
sodium channel, voltage-gated, type I, alpha	SCN1A	M22253	12	Plasma Membrane
purinergic receptor P2X-like 1	P2RXL1	X92070	-2	Plasma Membrane
gamma-aminobutyric acid A receptor gamma 2	GABRG2	X56313	-19	Plasma Membrane
				
**Kinases**				
G protein-coupled receptor kinase 5	GRK5	NM_005308	131	Plasma Membrane
protein kinase, cGMP-dependent, type II	PRKG2	Z36276	68	Cytoplasm
mitogen-activated protein kinase kinase kinase kinase 1	MAP4K1	Y09010	25	Cytoplasm
calcium/calmodulin-dependent serine protein kinase	CASK	U47110	3	Plasma Membrane
discs, large homolog 1	DLG1	U14950	3	Plasma Membrane
phosphatidylinositol 4-kinase beta	PI4KB	D84667	3	Cytoplasm
discoidin domain receptor family member 1	DDR1	L26525	-8	Plasma Membrane
non-metastatic cells 6	NME6	AF051943	-14	Extracellular Space
				
**Peptidases**				
carboxypeptidase A3	CPA3	U67914	5	Extracellular Space
ADAM metallopeptidase domain 17	ADAM17	AJ012603	4	Plasma Membrane
Proteasome subunit alpha 1	PSMA1	M29859	3	Cytoplasm
protein disulfide isomerase family A member 3	PDIA3	D63378	2	Cytoplasm
caspase 1	CASP1	U14647	-5	Cytoplasm
				
**Phosphatases**				
dual specificity phosphatase 6	DUSP6	U42627	53	Cytoplasm
protein phosphatase 1 subunit 1A	PPP1R1A	AJ276593	18	Cytoplasm
protein tyrosine phosphataser type 11	PTPN11	U09307	-2	Cytoplasm
				
**Transcription regulators**				
jun dimerization protein 2	JDP2	U53449	-2	Nucleus
cAMP responsive element modulator	CREM	Z15158	-4	Nucleus
				
**Transmembrane receptors**				
cholinergic receptor, nicotinic, beta 1	CHRNB1	X74833	39	Plasma Membrane
				
**Transporters**				
solute carrier family 1 member 1	SLC1A1	U21104	238	Plasma Membrane
solute carrier family 22, member 3	SLC22A3	AF055286	95	Plasma Membrane
gap junction protein, beta 2	GJB2	X51615	55	Plasma Membrane
solute carrier family 1, member 3	SLC1A3	S59158	6	Plasma Membrane
solute carrier family 22, member 6	SLC22A6	AF008221	6	Plasma Membrane
vacuolar protein sorting 33 homolog B	VPS33B	U35245	4	Cytoplasm
solute carrier family 30, member 1	SLC30A1	U17133	3	Plasma Membrane
syntaxin 4	STX4	L20821	2	Plasma Membrane
murinoglobulin 1	MUG1	J03552	-2	Extracellular Space
ATPase, Cu++ transporting, beta polypeptide	ATP7B	AF120492	-6	Cytoplasm
				
**Others**				
BCL2/adenovirus E1B interacting protein 3	BNIP3	AF243515	216	Cytoplasm
natriuretic peptide precursor C	NPPC	D90219	109	Extracellular Space
trans-golgi network protein 2	TGOLN2	X53565	106	Cytoplasm
fibrillin 2	FBN2	L39790	105	Extracellular Space
amyloid P component, serum	APCS	M83177	85	Extracellular Space
zinc finger, matrin type 3	ZMAT3	Y13148	84	Nucleus
LIM and senescent cell antigen-like domains 1	LIMS1	AAA20086	75	Plasma Membrane
CD44 molecule	CD44	U96138	61	Plasma Membrane
common salivary protein 1	LOC171161	U00964	54	Extracellular Space
selectin P	SELP	L23088	44	Plasma Membrane
collagen, type XI, alpha 1	COL11A1	AJ005396	39	Extracellular Space
collagen, type XII, alpha 1	COL12A1	U57362	28	Extracellular Space
nucleosome assembly protein 1-like 4	NAP1L4	AJ002198	22	Nucleus
spermine binding protein	SBP	J02675	20	Unknown
ribosomal protein L35	RPL35	M34331	6	Cytoplasm
connector enhancer of kinase suppressor of Ras 2	CNKSR2	AF102854	5	Plasma Membrane
prolactin family 8, subfamily a, member 81	PRL8A8	AB000107	4	Extracellular Space
extracellular proteinase inhibitor	EXPI	X13309	3	Extracellular Space
fibrinogen gamma chain	FGG	J00735	3	Extracellular Space
smooth muscle alpha-actin	ACTA2	X06801	2	Unknown
tropomyosin 1 alpha	TPM1	M34134	2	Cytoplasm
calcineurin binding protein 1	CABIN1	AF061947	-2	Nucleus
crystallin, gamma E	CRYGE	J00716	-2	Unknown
follistatin-like 1	FSTL1	M91380	-2	Extracellular Space
secreted phosphoprotein 2	SPP2	U19485	-2	Extracellular Space
tachykinin, precursor 1	TAC1	M15191	-2	Extracellular Space
myosin light chain 9	MYL9	S77900	-3	Cytoplasm
ubiquitin B	UBB	X51703	-3	Cytoplasm
golgin B1 protein	GOLGB1	D25543	-6	Cytoplasm
lysosomal-associated membrane protein 1	LAMP1	X14765	-11	Plasma Membrane

Gene description names, gene symbols are from IPA Tool; accession numbers are from GenBank

Only a small overlapping gene subset is shared by IL-6 and NGF comprising a total of 16 genes and including the major functional categories enzymes (3 genes), G-Protein coupled receptors (1), growth factors (1), ion channels (2), kinases (1), peptidases (2), transporters (1) and molecules with other functions (5; Table 3). All genes are regulated in a parallel fashion except for caspase 1 with an opposite expression pattern of IL-6 (40-fold) as compared to NGF (-5-fold; Table 3). Tables 1, 2, 3 summarize gene description names, Genbank accession numbers and changes in expression levels derived from the Chip analyses, gene symbols and abbreviations derived from the IPA Tool.

**Table 3:** Set of genes commonly regulated by IL-6 and NGF in PC12 cells

**Gene**		**Fold change**	
		**IL-6**	**NGF**
**Enzymes**			
guanine nucleotide binding protein, alpha z	GNAZ	14	9
glutathione S-transferase A3	GSTA3	- 3	- 3
aldo-keto reductase family 1, member C1	AKR1C1	12	10
			
**G-protein coupled receptors**			
cholinergic receptor, muscarinic 3	CHRM3	- 3	- 2
			
**Growth factors**			
growth differentiation factor 15	GDF15	80	131
			
**Ion channels**			
glutamate receptor, ionotropic, delta 2	GRID2	91	78
purinergic receptor P2X, ligand-gated ion channel	P2RX2	11	13
			
**Kinases**			
phosphatidylinositol 4-kinase beta	PI4KB	2	3
			
**Peptidases**			
caspase 1	CASP1	40	- 5
proteasome subunit alpha 1	PSMA1	5	3
			
**Transporters**			
syntaxin 4	STX4	3	2
			
**Others**			
trans-golgi network protein 2	TGOLN2	113	106
LIM and senescent cell antigen-like domains 1	LIMS1	94	75
fibrinogen gamma chain	FGG	94	3
collagen, type XII, alpha 1	COL12A1	26	28
extracellular proteinase inhibitor	EXPI	3	3

Gene description names, gene symbols are from IPA Tool

### Exemplary validation of microarray data using LightCycler quantitative RT-PCR analyses (qRT-PCR) on GAP-43 and REG3B mRNA expression

**Figure 1:**
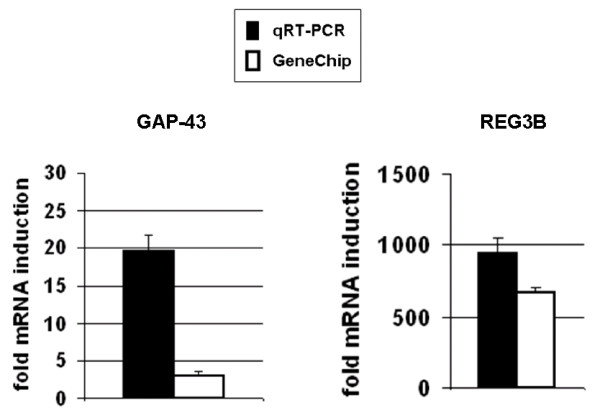
**Changes in expression of GAP-43- and REG3B mRNA levels in IL-6-stimulated PC12 cells determined by qRT-PCR versus GeneChip**. Affymetrix Genechip- and qRT-PCR analyses were performed as described in the Methods section.

**Figure 2:**
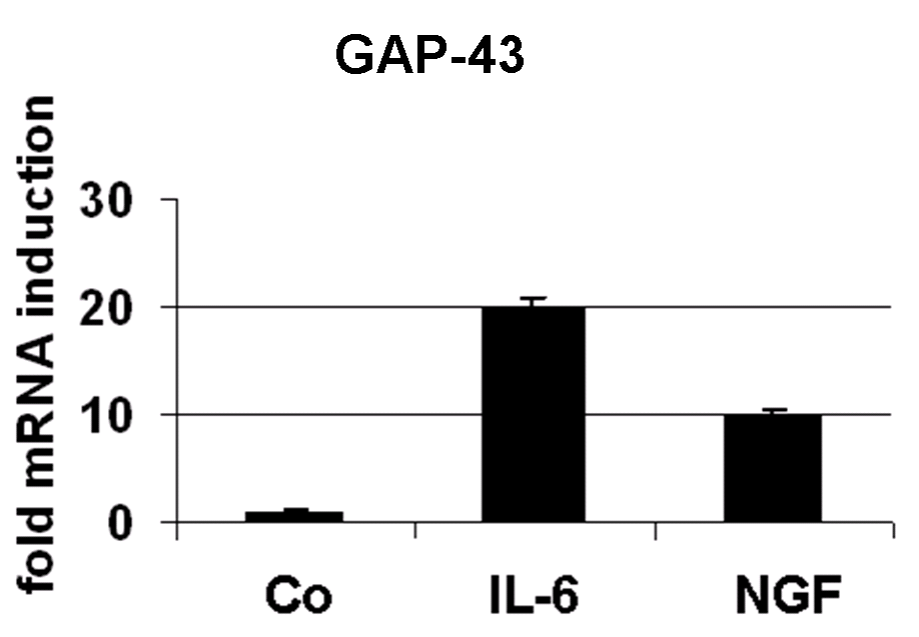
**Changes in expression of GAP-43- mRNA levels in IL-6- versus NGF-stimulated PC12 cells**. qRT-PCR analyses were performed as described in the Methods section.

For an exemplary validation of the microarray data, qRT-PCR using LightCycler was performed on GAP-43 and REG3B mRNA expression. In the microarray analyses, GAP-43 mRNA was found to be upregulated 3-fold by IL-6 (Table 1), whereas qRT-PCR revealed an induction of about 20-fold (Figure 1, left). In NGF-treated PC12 cells, GAP-43 mRNA was found to be upregulated by < 2-fold and therefore did not meet the exclusion criteria applied in the current work. However, qRT-PCR analyses revealed a 10-fold induction of GAP-43 mRNA levels induced by NGF in PC12 cells (Figure 2). Thus, PC12 cells treated with IL-6 or NGF undergo substantial neuronal differentiation. REG3B mRNA expression in the microarray analysis was found to be induced to 672-fold by IL-6 (Table 1), whereas qRT-PCR revealed an induction of REG3B mRNA by about 955-fold (Figure 1, right). In NGF-treated PC12 cells, neither microarray nor qRT-PCR analyses revealed changes in RGE3B expression.

### Ingenuity biological functional analyses of the gene sets regulated by IL-6 and NGF in PC12 cells

The criteria applied for the search of major biological function categories were maximum number of genes and the p-value of significance. As shown in Table 4, top biological functions found to be regulated by IL-6 include cancer (61 genes), cellular growth and proliferation (54 genes), cell death (47 genes), cell-to-cell signalling and interaction (46 genes), tissue development (45 genes) and others. A further gene set is involved in nervous system development and function (24 genes). The p-values in the range of 2.26 × 10^-7 ^to 3.77 × 10^-3 ^indicate statistical significance.

**Table 4:** Top high-level functions identified by Ingenuity global function analysis of regulated genes in IL-6-versus NGF- stimulated PC 12 cells

**Biological function classification**	**Number of genes**	**Significance (p-value)**
**IL-6-regulated genes**		
Cancer	61	2.98 × 10-6 to 5.16 × 10-3
Cellular Growth and Proliferation	54	1.14 × 10-6 to 5.16 × 10-3
Cell Death	47	4.54 × 10-6 to 5.16 × 10-3
Cell-to-Cell Signalling and Interaction	46	2.26 × 10-7 to 5.16 × 10-3
Tissue Development	45	2.26 × 10-7 to 5.15 × 10-3
Cellular Movement	39	9.19 × 10-6 to 5.16 × 10-3
Cellular Development	38	8.56 × 10-6 to 4.85 × 10-3
Small Molecule Biochemistry	37	1.32 × 10-5 to 4.47 × 10-3
...		
Nervous system development and function	24	2.83 × 10-5 to 3.77 × 10-3
		
**NGF-regulated genes**		
Cellular growth and proliferation	37	7.86 × 10-5 to 8.88 × 10-3
Cell-to-cell signalling and interaction	31	1.03 × 10-4 to 7.43 × 10-3
Molecular transport	30	8.89 × 10-6 to 8.70 × 10-3
Cancer	30	1.03 × 10-4 to 7.43 × 10-3
Cellular movement	29	2.41 × 10-5 to 8.70 × 10-3
Cell death	29	2.73 × 10-5 to 8.77 × 10-3
Neurological diseases	29	1.07 × 10-4 to 8.70 × 10-3
Nervous system development and function	29	1.60 × 10-4 to 8.70 × 10-3

p-values are from IPA Tool

Similarly, in NGF-treated PC12 cells top biological functions deal with the overall topics on cellular growth and proliferation (37 genes), cell-to-cell signalling and interaction (31 genes), molecular transport (30 genes), cancer (30 genes), cellular movement (29 genes) and others. One gene set is involved in nervous system development and function (29 genes). The p-values in the range from 8.89 × 10^-6 ^to 7.43 × 10^-3 ^indicate statistical significance (Table 4).

More detailed analyses for functional sub-categories are summarized in Table 5. Both stimuli utilize different repertoires of genes to exert the same biological functions that are all crucial for neuronal differentiation and nervous system development. Among others, important functional sub-categories include cellular growth (IL-6, 33 genes; NGF, 24 genes), differentiation (IL-6, 45 genes; NGF, 16 genes), cell movement (IL-6, 39 genes; NGF, 27 genes), chemotaxis (IL-6, 13 genes; NGF, 13 genes), adhesion of cells (IL-6, 26 genes; NGF, 18 genes), cellular signalling and small molecule biochemistry aiming at changing intracellular concentrations of second messengers such as Ca^2+ ^(IL-6, 16 genes; NGF, 16 genes) as well as cAMP (IL-6, 12 genes; NGF, 9 genes) as well as expression of posttranslational processing enzymes (IL-6, 23 genes; NGF, 15 genes). Table 5 (bottom) summarizes genes involved in specialized sub-categories of nervous system and development as far as they are represented in the IPKB.

**Table 5:** Ingenuity biological function analyses of IL-6-versus NGF-regulated genes in PC12 cells (selected)

	**IL-6 regulated genes in PC12 cells**		**NGF-regulated genes in PC12 cells**	
**Category**	**p-value**	**Molecules**	**p-value**	**Molecules**
Sub-Category or Function annotation				
**Cellular Growth and Proliferation**				
Growth of cells	2.27 × 10-4	ACVR2A, AHSG, ANXA1, BCL2L11, BRCA1, CASP1, CDC42, CHRM3, CXCL10, EGR1, FGFR1, GAP43, GDF15, GHR, GRID2, HGF, IGF2R, IRF1, ITGA7, JAK2, MAP2K5, MST1, MT3, MX1, NOS3, NOS2A, PIM3, RARA, SCAMP2, SDC2, STAT1, TIMP1, VDR	8.82 × 10-3	ACHE, AGTR1, BDNF, BNIP3, CASP1, CD44, CHRM3, CREM, DDR1, DUSP6, FBN2, FN1, GDF15, GJB2, GRID2, MYL9, NRG1, PDIA3, PTPN11, SLC30A1, TGFB1, TPM1, VPS33B, ZMAT3
Proliferation of cells	9.06 × 10-7	ACVR2A, ADCYAP1, ANXA1, AVP, BCL2L11, BRCA1, CALCB, CDC42, CHRM3, CHRM4, CRYAB, CXCL10, EGR1, FGFR1, FRK, GDF15, GHR, GHRH, HGF, IGF2R, IRF1, JAG2, JAK2, KLF6, KLK8, LCN2, MAP2K5, MT3, NFIB, NOS3, NOS2A, NR3C2, PDGFA, RARA, REG1A, REG3A, RNMT, SDC2, ST6GAL1, STAT1, TIMP1, TPM3, USF1, VDR, VIPR1	3.82 × 10-3	AGTR1, AKR1C3, BDNF, CALCA, CD44, CHRM3, DDR1, FN1, GDF15, GRK5, NPPC, NRG1, PPIA, PTPN11, TAC1, TGFB1
**Cellular Movement**				
Cell movement	2.18 × 10-8	ADCYAP1, ANXA1, CASP1, CDC42, CHRM3, CHRM4, CXCL10, CXCL13, EGR1, FCGR2A, FER, FGB, FGFR1, GNAZ, GRID2, HGF, HLA-G, HSP90AB1, IGF2R, JAK2, LCN2, LGALS9, LIMS1, MAP2K5, MST1, NOS3, NOS2A, OLR1, PDGFA, RARA, REG3A, SDC2, ST6GAL1, STAT1, TIMP1, TPM3, TUBB, VDR, VIPR1	7.96x10-5	ADAM17, AGTR1, APCS, BDNF, CALCA, CASP1, CD44, CHRM3, DDR1, FN1, GJB2, GNAZ, GRID2, LCAT, LIMS1, NAP1L4, NPPC, NRG1, PDE4B, PPIA, PTPN11, SCN2B, SELP, SLC1A3, TAC1, TGFB1, TPM1
Chemotaxis	4.05 × 10-4	ANXA1, CDC42, CXCL10, CXCL13, FCGR2A, FER, FGFR1, GNAZ, HGF, IGF2R, LGALS9, NOS3, VIPR1	6.29x10-5	AGTR1, BDNF, CALCA, CD44, FN1, GNAZ, NAP1L4, PDE4B, PPIA, PTPN11, SCN2B, TAC1, TGFB1
**Cell-To-Cell Signaling and Interaction**				
Adhesion of cells	1.47 × 10-7	ANXA1, CDC42, CDH17, CXCL10, EGR1, FCGR2A, FER, FEZ1, FGB, FGFR1, FGG, GRID2, HGF, IGF2R, ITGA7, JAG2, LGALS9, LIMS1, NOS3, OLR1, REG3A, SDC2, ST6GAL1, STAT1, STX4, TIMP1	1.34x10-4	ACHE, ADAM17, CASK, CD44, CNTN4, DDR1, DLG1, FGG, FN1, GRID2, LIMS1, NRG1, PTPN11, SELP, STX4, TAC1, TGFB1, TPH1
**Cell Signaling**				
Quantity of calcium	3.25 × 10-3	ADCYAP1, AVP, CHRM3, CXCL10, CXCL13, FCGR2A, GHRH, HGF, NOS3, NOS2A, VDR	8.89x10-6	AGTR1, BDNF, CALCA, CHRM3, FN1, GRK5, NPPC, PLD2, PPIA, PTHR1, PTPN11, SELP, TAC1, TGFB1
Production of nitric oxide	1.33 × 10-3	IRF1, JAK2, MST1, NOS3, NOS2A, STAT1	-	-
Flux of calcium	1.67 × 10-3	ADCYAP1, ANXA1, AVP, CHRM3, CXCL10, CXCL13, FCGR2A, P2RX2	2.20x10-3	CALCA, CHRM3, FN1, NPPC, P2RX2, PPIA, TGFB1
Cell surface receptor linked signal transduction	1.45 × 10-3	ACVR2A, ANXA1, CDC42, CXCL10, FCGR2A, FGFR1, ITGA7, JAK2, KLF6, LIMS1, PDGFA, PTPRD, STAT1	-	-
**Small Molecule Biochemistry**				
Quantity of cyclic AMP	1.00 × 10-5	ADCYAP1, AVP, CHRM4, CXCL10, GAP43, GHRH, GNAZ, NOS3, VIPR1	6.03x10-3	BDNF, CALCA, GNAZ, NPPC, PTHR1
Production of cyclic AMP	2.17 × 10-4	ADCYAP1, AVP, GHRH, GNAZ, NOS3, NOS2A, VIPR1		
Accumulation of cyclic AMP	1.21 × 10-3	ADCYAP1, AVP, AVPR2, CHRM3, GHRH, VIPR1	4.35 × 10-4	CALCA, CHRM3, GRK5, PTHR1, TAC1, TGFB1
Formation of cyclic AMP	1.28 × 10-4	ADCYAP1, AVP, AVPR2, GHRH, GANZ	7.26 × 10-4	CALCA, GNAZ, PTHR1, TAC1
Release of Ca2+	9.82 × 10-5	ANXA1, AVP, CHRM3, FCGR2A, FGB, FGG	-	-
Quantity of cholesterol	-	-	2.85 × 10-3	ATP7B, BDNF, CALCA, GULO, LCAT
**Post-Translational Modification**				
Modification of protein	1.57 × 10-5	AVP, BRCA1, CASP1, CHRM3, FCGR2A, FER, FGFR1, GRID2, HSP90AB1, HTATIP, JAK2, LHB, MST1, NOS3, NOS2A, PDGFA, PDIA2, PDP2, PIM3, PTPRD, ST6GAL1, STAT1, TGM1	4.47 × 10-3	APCS, CASP1, CD44, CHRM3, DUSP6, FN1, GRID2, NDST1, NRG1, PDIA3, PPIA, PTPN11, RABGGTA, TAC1, UBB
**Nervous system development and function**				
growth of neurites	8.02 × 10-3	ADCYAP1, CDC42, GAP43, HGF, TPM3	-	-
survival of neurons	3.60 × 10-3	ADCYAP1, BCL2L11, GDF15, HGF, RARA, REG3A	-	-
development of synapse	6.57 × 10-3	GRID2, NFASC	-	-
fasciculation of axons	3.14 × 10-2	GAP43	-	-
complexity of dendritic trees	1.25 × 10-2	HGF	-	-
long-term potentiation of dentate gyrus	1.25 × 10-2	EGR1	-	-
neurological process of synapse	-	-	1.60 × 10-4	BDNF, CHRM3, CHRNB1, NRG1, PPP1R1A
synaptic transmission	-	-	2.88 × 10-4	BDNF, CACNB2, CHRM3, CHRNB1, GABRG2, P2RX2, SCN2B, SLC1A1, SLC1A3
neurological process of axons, neurites	-	-	4.79 × 10-4	BDNF, CNTN4, GRID2, NRG1, PDIA3, UBB
activation of nerves	-	-	7.73 × 10-4	CALCA, TAC1
binding of neurites	-	-	7.73 × 10-4	BDNF, CD44
size of cell body	-	-	7.73 × 10-4	ACHE, BDNF
survival of neurons	-	-	8.92 × 10-4	BDNF, GDF15, NRG1, PDIA3, SLC1A3, TGFB1
development of neurites	-	-	2.83 × 10-3	ACHE, BDNF, GRID2, NRG1, PDIA3, PTPN11
migration of nervous tissue cell lines	-	-	3.38 × 10-3	NRG1, TGFB1
proliferation of nervous tissue cell lines	-	-	6.67 × 10-3	NPPC, TGFB1

-, no subcategories found in IPA Tool; p-values and gene symbols are from IPA Tool

## Discussion

In a previous study, we have used PC12 cells to examine the effects of IL-6/s-IL6R on neuronal differentiation in comparison to NGF [22]. Already after 24 hours of exposure to IL-6/s-IL-6R or NGF PC12 cells are highly active in cellular growth and proliferation displaying pronounced formation of extending neurites. Combined incubation with IL-6/s-IL-6 plus NGF drastically enhanced cell number and neurite outgrowth arguing for an additive effect of both stimuli on neuronal differentiation. In the current study we have chosen this time point to perform microarray analyses in order to monitor changes in gene expression and to compare the genetic programs utilized for neuronal differentiation by IL-6 versus NGF.

An important aspect in gene expression profiling using microarrays is the accuracy of the measurements in the relative changes in mRNA expression. Thus, alternative technologies such as qRT-PCR are used for the validation of microarray data [27]. Several systematic studies comparing the changes in gene expression obtained from oligonucleotide- or cDNA arrays to data from qRT-PCR revealed that a good correlation exists for genes exhibiting fold-change differences in expression of > 2-fold [28,29]. Therefore, in our datasets all genes displaying changes in expression levels of < 2-fold were excluded. Moreover, our exemplary validation data on GAP-43- and REG3B-expression are in line with other previous reports confirming that it is rather the magnitude of fold change varying between qRT-PCR and Affymetrix-analysis, but not the direction.

Detailed Ingenuity biological function analyses reveal that IL-6 and NGF activate gene sets that regulate the same process in neuronal differentiation and nervous system development, however, utilizing completely distinguished sets of individual molecules. This may explain our previous observation that combined application of IL-6/s-IL-6R plus NGF generates an additive effect on PC12 cell differentiation. Important processes in neuronal differentiation and nervous tissue development include cellular growth and proliferation in order to enhance cell number. Neurite outgrowth and network generation requires migration of neurons or nerve growth cones. Neuronal navigation is guided by the interaction of the neuron with its local environment, in particular by chemotaxis as the key mechanism. This process involves three major steps including directional sensing along a gradient of chemotactic factors, cellular motility i.e. the cell's movement by changes in cytoskleletal organisation and cellular adhesion and cellular polarisation [30-32]. Certainly, a key step in the regulation of these processes is the increased gene expression of growth factors and functionally related external molecules, indicating convergence of several different signaling pathways (Table 5). In IL-6 stimulated PC12 cells these tasks may be taken by growth differentiation factor 15 (GDF15), platelet-derived growth factor alpha (PDGFA), hepatocyte growth factor (HGF), regenerating islet-derived 3 alpha (REG3A), regenerating islet-derived 3 beta/pancreatitis-associated protein I (REG3B/PAPI), growth hormone releasing hormone (GHRH) and adenylate cyclase activating polypeptide (PACAP). NGF recruits GDF15 (131-fold), transforming growth factor beta 1 (TGFB1; 101-fold) and brain-derived neurotrophic factor (BDNF; 89-fold). TGFB1 is the prototype member of the TGFB-superfamily comprising multifunctional growth factors with numerous cell and tissue functions such as cell cycle control, regulation of early development, differentiation, extracellular matrix (ECM) formation and chemotaxis. In the nervous system, TGFB1 has been shown to regulate neuroprotection against glutamate cytotoxicity, ECM production, and cell migration in the cerebral cortex, control of neuronal death as well as survival of neurons (reviewed in [33]). GDF15 is a member of the TGFB- superfamily and has been shown to be a potent trophic factor in the brain (reviewed in [34]). Hepatocyte growth factor (HGF) is a chemoattractant and a survival factor for embryonic motor neurons. In addition, sensory and sympathetic neurons and their precursors respond to HGF with increased differentiation, survival and axonal outgrowth [35]. Moreover, HGF may synergize with other neurotrophic factors to potentiate the response of developing neurons to specific signals [36]. Platelet derived growth factor (PDGF) has been suggested to support neuronal differentiation [37], and has previously been reported to act as a mitogen for immature neurons [38] and neural progenitor cells [39]. REG3A and REG3B/PAPI are members of the regenerating protein (REG)/pancreatitis-associated protein (PAP) family representing a complex group of small secretory proteins which display many different functions, among them growth factor activity for neural cells [40]. So far, only limited knowledge is available about the role and function of PAP/REG-proteins in the nervous system. REG3B/PAPI expression is induced in spinal motor neurons as well as subsets of the dorsal root ganglion neurons [41]. Moreover, *in vitro *REG3B/PAPI has a mitogenic effect on Schwann cells [42]. In a hypoglossal nerve injury model in rats, expression of REG3B/PAPI mRNA was found to be enhanced in injured motor neurons after axotomy and a marked induction of REG3G/PAPIII mRNA was observed in the distal part of the injured nerve [43]. More recently, REG3G/PAPIII has been identified as a macrophage chemoattractant that is induced in and released from injured nerves [44]. With REG1A/PSP and REG3G/PAPIII, two further members of the REG/PAP family are induced by IL-6 in PC12 cells. It is noteworthy that these genes are up-regulated at the highest levels obtained in the entire dataset for IL-6. In NGF-treated PC12 cells, no up-regulation of the PAP/REG protein genes was observed. The results in our study are in line with an earlier report demonstrating up-regulation of PAP/REG gene family members in PC12 cells upon stimulation with IL-6/s-IL-6R [45].

So far various studies have investigated gene expression profiles in NGF-treated PC12 cells applying different experimental protocols in respect to time points and periods of NGF administration [46-51]. From most studies, it is obvious that PC12 cells require at least 3 to 5 days of NGF-treatment to obtain the fully differentiated neuronal phenotype. The most significant morphological changes occur within the first 2 days, reaching a plateau phase at day 3 [51]. Redundant data sets as well as unique genes have been identified and followed. Our study provides novel candidate genes activated in the early phase of the differentiation process and thus may enlarge the repertoire of known NGF-regulated genes.

The current study reveals novel aspects of IL-6 action, notably that it applies several major routes to direct PC12 cell differentiation. Besides up-regulation of growth factors known to act in autocrine and paracrine fashion to take over further tasks in the differentiation process, these include induction of PACAP, a pleiotropic molecule with a broad spectrum of biological functions. Among them are actions as a neurotrophic factor similar to NGF as well as induction of transcription factors known to be of key importance in neuronal differentiation [52].

Upregulation of PACAP could have an important impact on IL-6-induced PC12 cell differentiation. A recent report provided data from microarray analyses of PACAP-regulated gene transcripts in primary cultures of sympathetic neurons at 6 hours and 92 hours of stimulation [53]. A comparison with our data reveals that many gene families that are activated by PACAP in primary sympathetic neurons are also induced by IL-6 in PC12 cells (Table 6). Thus, many of the effects of IL-6 on PC12 cells are likely to be mediated by the intermediate autocrine and/or paracrine action of PACAP. PACAP is a member of a family of neuropetides known to activate class II G-protein coupled receptors (GPCRs; reviewed in [54]). Other family members include growth hormone releasing hormone (GHRH) and calcitonin-related peptide beta (CALCB) which are activated by IL-6 in PC12 cells by 31-and 195-fold, respectively. All members of the class II GPCR superfamily regulate intracellular cAMP-levels by receptor coupling to the Gs-adenylate cyclase-cAMP signaling pathway [54]. A further mechanism of PACAP action in PC12 cells could be a transactivation of TrkA receptors [55]. However, in light that the overlap in the datasets of IL-6 versus NGF is rather small, TrkA activation may not be a primary event at all or at the time point of our study.

**Table 6:** Comparison of commonly regulated gene families in PACAP-stimulated sympathetic primary neurons versus IL-induced PC12 cells (data derived from [53])

**PACAP-stimulated sympathetic neurons (data are from **[53]**)**			**IL-6-stimulated PC12 cells**	
**Gene family**				
Gene abbreviation	**9 hours**	**96 hours**	Gene abbreviation	**24 hours**
**Pituitary adenylate cyclase activating polypeptide**				
ADCYAP1	+	+	ADCYAP1	+
				
**BCL2-like protein**				
BCL2L11	+	n.c.	BCL2L11	+
				
**Chemokine Ligands**				
CXCL1	+	+		
			CXCL10	+
			CXCL13	+
				
**Cytochrome P450 proteins**				
CYP1B1	+	+		
			CYP4F16	+
**Early growth response**				
EGR1	+	n.c.	EGR1	+
				
**Glutathione S-transferase**				
GSTA3	+	n.c.	GSTA3	-
				
**Heat shock proteins**				
HSP27B1	+	n.c.	HSP90B1	+
				
**Janus kinase**				
JAK2		+	JAK2	+
				
**Kruppel-like factors**				
KLF4	+	n.c.	KLF6	+
KLF9	+	n.c.		
				
**Nuclear factors**				
NFIA	+	n.c.	NFIB	+
				
**Nuclear receptors**				
NR4A3	+	n.c.		
NR4A2	+	n.c.		
NR4A1	+	n.c.		
			NR3C2	+
				
**Sialytransferases**				
ST8SIA1	+	+	ST8SIA3	-
ST6GAL1	+	+	ST6GAL1	+
				
**Solute carrier proteins**				
SLC1A3	+	n.c.		
SLC2A1		+		
SLC2A3	+	+		
			SLC6A3	+
SLC7A1	+	+		
SLC7A3		+		
			SLC12A5	-
SLC18A2	+	+		
			SLC30A2	-
SLC24A2		+		
				
**Tubulins**				
TUBA1	-	n.c.		
			TUBB	+
				
**Tissue Inhibitor of metalloproteinase**				
TIMP1	+	+	TIMP1	+

+, upregulated -, downregulated; n.c., not changed from control cultures; gene symbols are from IPA Tool

A further key step in IL-6 actions on PC12 cell differentiation is the induction of RARA and EGR-1/Zif268, two transcription factors known to be of crucial importance in neuronal differentiation. Among the genes regulated by retinoic acid is GAP-43, a neuron specific protein frequently used as a marker of neuronal differentiation as it is expressed in most neurons during neuronal development, nerve regeneration and LTP [56-60]. The data herein are confirmative to our previous study in which we have found induction of GAP-43 mRNA upon stimulation of PC12 cells with IL-6/s-IL-6R [22]. EGR-1/Zif268 is induced in nearly every model of long-lasting synaptic plasticity in the CNS [61-64] and suppression of Zif268 prevents neurite outgrowth in PC12 cells [65]. Recently candidate target genes of Zif268 in PC12 cells were identified suggesting that a key component of the long-lasting effects of Zif268 on CNS plasticity is the regulation of proteasome activity [66,67].

Signal transducer and activator of transcription 1/2 (STAT1/2), two members of the STAT family of transcriptions factors involved in signaling by Interferons (IFN) [68] are activated by stimulation of the PC12 cells with IL-6. As we could not detect changes in IFN gene expression, an autocrine action of PDGF is the most likely candidate for upregulation of STAT1/2 as described for neural progenitor cells [39]. STAT1/2 may upregulate interferon regulatory factor 1(IRF1)-expression, a further transcription factor of IFN-signaling. Breast cancer 1 (BRCA1) encodes a tumour suppressor gene whose germ line mutations in women are associated with a genetic predisposition to breast and ovarian cancer. STAT1 transcriptional activity is decreased by a physical interaction with BRCA1 as a key step in the regulation of IFN-induced cellular growth arrest [69]. By the action of IL-6, BRCA1 gene expression is down-regulated thus supporting STAT1 mediated PC12 cell growth. We failed to detect STAT3 expression, the key transcription factor of IL-6 signaling. This is most likely due to the fact that STAT3 gene transcription occurs very early in IL-6-stimulation and is already terminated at the time point of the analysis, or the expression levels are below 2-fold and thus did not meet the exclusion criteria.

The morphological changes during nervous system development are controlled by interactions of individual neurons with the ECM. Signals from the ECM into a particular neuron are mediated by integrins via associated adapter molecules. In this way growth factor induced receptor tyrosine kinase (RTK)- and integrin-mediated signalling determine the fate of a particular cell, notably differentiation, cell shape, adhesion, polarity, migration, as well as proliferation versus apoptotic cell death (reviewed in [70]). LIM and senescent cell antigen-like domains1/PINCH (LIMS1/PINCH) is an intracellular adaptor molecule providing the molecular link of an integrin-RTK network. LIMS1 physically connects integrin-linked kinase (ILK) to non-catalytic (region of) tyrosine kinase adaptor protein 2 (Nck2), an adapter molecule of the growth factor receptor (RTK) [70]. LIMS1 is activated by IL-6 as well as NGF and thus is one of few genes regulated in the common subset. In contrast to IL-6, NGF simultaneously up-regulates major components of the ECM including collagen, type XI, alpha1 (COL11A1), COL12A1, fibronectin1 (FN1) as well as fibrillin2 (FN2) (Table 2).

In contrast to NGF, only one publication provided expression profiling data analysing gene sets regulated by IL-6 upon neuronal differentiation. Primary cultures of rat dorsal root ganglia (DRG) were treated with IL6RIL6 for 2 and 4 days, respectively. A detailed comparison reveals that only a small number of commonly regulated genes may be identified in the datasets that are regulated in parallel or opposite direction. These include Egr-1 (upregulated in PC12 cells; downregulated in DRG cells), TGFA (upregulated in PC12 cells and DRG cells), TGFB (upregulated in PC12 cells; downregulated in DRG cells), PDGFA (upregulated in PC12 cells; downregulated in DRG cells) and IRF-1 (upregulated in PC12 cells and in DRG cells) [24].

The results obtained from our study may also have impact into clinical treatments of injured peripheral nerves which, in contrast to central nerves, have the ability to recover from damage. Currently the therapy of choice is the use of autologous grafts where the defect is bridged with a section of autologous nerve tissue, mostly a sensory nerve [71]. Alternatively, nerve conduits or decellularized nerve grafts can be used; however, no therapy could yield a satisfactory functional recovery [72]. Various combinations of NTs, neuropoietic cytokines and GFLs have been shown to generate a microenvironment suitable to improve nerve repair [26]. The results of our study may provide novel aspects for the treatment of peripheral nerve injury as the local application of a designer cytokine such as H-IL-6 with a strongly enhanced bioactivity on neuronal development and neurite outgrowth in combination with NTs and/or GFLs may create a microenvironment with a strong reparative potency.

## Conclusion

IL-6 and NGF utilize different genetic programs to exert the same biological functions in neuronal differentiation. An important step is the recruitment of many growth factors that may act in autocrine and/or paracrine fashion and may control the long-term effects on growth, neuronal differentiation or survival.

## Methods

### Reagents, buffers and cells

DMEM medium, horse serum, fetal bovine serum and other cell culture supplements were obtained from GibcoBRL. TRIZOL reagent and Superscript reverse transcriptase were purchased Life Technologies. PC12 cells were obtained from ATCC, Manassas (VA), USA. Hyper-IL-6 was generated as described [8]. The LightCycler PCR kit was from Roche Diagnostics, Mannheim, Germany.

### Cell culture

PC12 cells were cultured in DMEM medium containing 10% fetal bovine serum and 100 U/ml penicillin and streptomycin at 37°C in humidified 5% CO_2_/95% air. For stimulation confluent cells were washed once with PBS and cultured in cell culture medium containing 10 ng/ml H-IL-6 or 50 ng/ml recombinant human NGF for 24 hours. Control cells were incubated in cell culture medium alone for 24 hours.

### RNA Preparation

Total RNA from unstimulated (control), H-IL-6- and NGF- stimulated PC12 cells was isolated using TRIZOL reagent according to the manufacturer's instructions. RNA was quantified spectrophotometrically by measuring the absorbance at 260 nm and the integrity was checked by formaldehyde agarose gel electrophoresis. The extracted RNA was stored at -80°C.

### GeneChip analysis

20 μg of total RNA was used for each experiment and the target cRNA for Affymetrix Gene Chip analysis was prepared according to the manufacturer's instructions. Affymetrix GeneChip Rat Genome U34A arrays containing each 8'799 probes including full-length or annotated rat genes and several thousands of rat EST clusters consisting of redundant probes spanning an identical transcript were hybridized with the target cRNAs at 45°C for 16 h, washed and stained by using the Gene Chip Fluidics Station. The arrays were scanned with the Gene Array scanner (Affymetrix), and the fluorescence images obtained were processed by the Expression Analysis algorithm in Affymetrix Microarray Suite (ver. 4.0) and Microsoft Excel. Data were imported into GeneSpring^® ^analysis software (ver. 4.1.3, Silicon Genetics, Redwood City, CA) for further analysis. Genes that showed substantial up- or down-regulation after stimulation by fold changes > 2 were selected from three independent experiments. Genes whose fold change was < 2 and expressed sequence tags (ESTs) that were not fully identified were excluded from the gene list. Thus, only genes with a change fold cutoff > 2 were considered to be significantly differentially regulated. Values are given as round off numbers. For each condition (unstimulated control- and H-IL-6-simulated PC12 cells or unstimulated control and NGF-stimulated PC12 cells) 3 independent microarray analyses (n = 3) were performed using RNA samples derived from independently prepared cell culture batches.

### Quantitative Real Time PCR (qRT-PCR)

Total RNA (10 μg) from individual samples cultured separately from those used for microarray analyses was reverse-transcribed using Superscript II Reverse Transcriptase (GibcoBRL) according to the manufacturer's instructions.

PCR reactions were performed in glass capillaries with the LightCycler thermal cycler system (Software version 3.5; Roche Diagnostics, Mannheim, Germany) using the LightCycler DNA Master SYBR Green I kit (Roche Diagnostics, Mannheim) according to the manufacturer's instructions. The primers used for RT-PCR analyses were rat S12 forward: 5'-GGC ATA GCT GCT GGA GGT GTA A-3'; rat S12 reverse: 5'-CCT TGG CCT GAG ATT CTT TGC-3'; rat REG3B forward: 5'-GGT TTG ATG CAG AAC TGG CCT-3'; rat REG3B reverse: 5'-TGA CAA GCT GCC ACA GAA TCC-3'; rat GAP-43 forward: 5'-CGT TGC TGA TGG TGT GGA GAA-3'; rat GAP-43 reverse: 5'-GCA GGC ACA TCG GCT TGT TTA-3'. PCR conditions were: 50 cycles with denaturation at 95°C for 8 seconds, annealing at 57°C for 8 seconds, and extension at 72°C for 14 seconds. Negative controls without cDNA (non-template controls; ntc) were run concomitantly. Specificity of amplified PCR products was confirmed by melting curve analysis after completion of the PCR run. Each PCR was performed in 3 independent experiments (n = 3) using different cell-culture batches.

### Quantification of LightCycler qRT-PCR data

Quantification of data was performed with the LightCycler software 3.3 (Roche Diagnostics) using the ΔΔC_p _method. The difference between the crossing points (CPs; ΔC_p _values) for the target mRNA samples and reference S12 RNA samples (ΔΔC_p_) was used to calculate the expression values of the target mRNAs (2^-Δ(ΔCp)^).

### Ingenuity global functional analyses

To investigate possible biological interactions of differently regulated genes, datasets representing genes with altered expression profile derived from microarray analyses were imported into the Ingenuity Pathway Analysis Tool (IPA Tool; Ingenuity^®^Systems, Redwood City, CA, USA; ). The basis of the IPA-program consists of the Ingenuity Pathway Knowledge Base (IPKB) which is derived from known functions and interactions of genes published in the literature. Thus, the IPA Tool allows the identification of biological networks, global functions and functional pathways of a particular dataset. The complete dataset containing gene identifiers (Genbank accession numbers) and corresponding expression values was uploaded into the application. Each gene identifier is mapped to its corresponding gene object in the IPKB. Each gene product is assigned to functional (e.g. "cellular growth and proliferation") and sub-functional (e.g. "colony formation") categories. The biological functions that are most significant to the dataset are identified by the use of Fischer's exact test to calculate a p-value that determines the probability that each biological function assigned to that data set is due to chance alone.

### Statistical analysis

Differences were tested by Welch's t-test based on three independent experiments, and p-values less than 0.05 were considered statistically significant. Values are expressed as means ± SEM.

## Authors' contributions

DK and GW generated the microarray data and drafted the manuscript. UC provided the microarray facility. MB performed the statistical analyses of the microarrays. BD and PM performed the cell-culture of PC12 cells. DK and UO provided support, direction and oversight of the experiments and revised the final manuscript. UO holds the SNF grant.
